# PRDX6 Inhibits Neurogenesis through Downregulation of WDFY1-Mediated TLR4 Signal

**DOI:** 10.1007/s12035-018-1287-2

**Published:** 2018-08-10

**Authors:** In Jun Yeo, Mi Hee Park, Dong Ju Son, Ji Young Kim, Kyoung Tak Nam, Byung Kook Hyun, So Young Kim, Myung Hee Jung, Min Ji Song, Hyung Ok Chun, Tae Hyung Lee, Sang-Bae Han, Jin Tae Hong

**Affiliations:** College of Pharmacy and Medical Research Center, Chungbuk National University, 194-31, Osongsaengmyeong 1-ro, Heungdeok-gu, Cheongju, Chungbuk 361-951 Republic of Korea

**Keywords:** PRDX6, WDFY1, TLR4, Neurogenesis

## Abstract

Impaired neurogenesis has been associated with several brain disorders, such as Alzheimer’s disease (AD) and Parkinson’s disease (PD). The role of peroxiredoxin 6 (PRDX6) in neurodegenerative diseases is very controversial. To demonstrate the role of PRDX6 in neurogenesis, we compared the neurogenesis ability of PRDX6-overexpressing transgenic (Tg) mice and wild-type mice and studied the involved molecular mechanisms. We showed that the neurogenesis of neural stem cells (NSCs) and the expression of the marker protein were lower in PRDX6 Tg-mice than in wild-type mice. To determine the factors involved in PRDX6-related neural stem cell impairment, we performed a microarray experiment. We showed that the expression of WDFY1 was dramatically decreased in PRDX6-Tg mice. Moreover, WDFY1 siRNA decreases the differentiation ability of primary neural stem cells. Interestingly, WDFY1 reportedly recruits the signaling adaptor TIR-domain-containing adapter-inducing interferon-β (TRIF) to toll-like receptors (TLRs); thus, we showed the relationship among TLRs, PRDX6, and WDFY1. We showed that TLR4 was dramatically reduced in PRDX6 Tg mice, and reduced TLR4 expression and neurogenesis was reversed by the introduction of WDFY1 plasmid in the neural stem cells from PRDX6 Tg mice. This study indicated that PRDX6 inhibits the neurogenesis of neural precursor cells through TLR4-dependent downregulation of WDFY1 and suggested that the inhibitory effect of PRDX6 on neurogenesis play a role in the development of neurodegenerative diseases in the PRDX6 overexpressing transgenic mice.

**Electronic supplementary material:**

The online version of this article (10.1007/s12035-018-1287-2) contains supplementary material, which is available to authorized users.

## Introduction

Neurogenesis is impaired in neurodegenerative diseases, such as Alzheimer’s disease (AD) and Parkinson’s disease (PD) [1–3]. Extensive evidences indicate that the promotion of neurogenesis resolves the symptoms of the diseases [4, 5]. It has been demonstrated that anti-amyloid immunotherapy against β-amyloid (Aβ) promotes recovery from AD and neurotoxicity by restoring neuronal population [4]. Tchantchou et al. also demonstrated that the administration of EGb 761 helped recover memory function by enhanced neurogenesis in the hippocampus of Tg2576 AD mouse model [5]. In addition, lithium improves hippocampal neurogenesis and thus recovers cognitive functions in (amyloid precursor protein) APP mutant mice [6]. However, deficits of neural stem cell differentiation are associated with the development of neurodegenerative diseases. Familial AD (FAD)-linked mutations in APP transgenic mice led to impaired neurogenesis. Presenilin 1 (PS1) FAD mutant transgenic mice also showed impaired neural precursor cell proliferation and survival [7, 8]. The double-transgenic mice that expresses APP (KM670/671NL)/PS1 (Δexon9) and mutant APPswe/PS1DeltaE9 in neurons showed neuritic dystrophy [9] and impairments in neurogenesis [10]. Further, PD-associated leucine-rich repeat kinase 2 (LRRK2) mutation reportedly inhibits neuronal differentiation of neural stem cells [11]. PTEN-induced putative kinase 1 (PINK1)-deficient mice also show decreased brain development and neural stem cell differentiation [12]. A recent study also demonstrated that α-synuclein (SNCA) gene triplication in PD impairs neuronal differentiation and maturation of PD patient-derived-induced pluripotent stem (iPS) cells [13]. Moreover, a recent study demonstrated that proliferating cells do not become mature neurons in the brain of AD patients [14], and compared with controls, the brains of PD patients had fewer proliferating cells [15]. These results suggested that neurodegenerative diseases, including AD and PD, are associated with impaired neurogenesis.

Cell differentiation have shown marked correlation with cellular oxygen species levels in the nervous system. Oxidative stress is a deleterious condition leading to cellular death, and it plays a key role in the development and pathology of neurodegenerative diseases through impaired neurogenesis. In the pathophysiology of neurodegenerative diseases like PD and AD [16], as well as that of other conditions, such as ischemic stroke, a state of oxidative stress accompanied with altered neurogenesis has been implicated [17]. Antioxidants reduce the deleterious activity of oxidative stress and potentially delay the development of neurodegenerative diseases; thus, they are very important for prevention of neurodegenerative diseases. Recent findings showed that when examined at 3–4 months of age, genetic deficiency of extracellular superoxide dismutase (SOD) was associated with significantly suppressed baseline neurogenesis and impaired hippocampus-dependent cognitive function [18]. Other studies also demonstrated that the lack of extracellular SOD in the microenvironment impacts radiation-induced changes in neurogenesis [19]. Antioxidant proteins like metallothionein are reported to help recuperation from brain injury by increasing neurogenesis [20]. Metallothionein-null mice display impaired brain parenchyma recovery following injury, whereas metallothionein overexpression in transgenic mice and exogenous administration of metallothionein in mice showed relatively less damage owing to enhanced neurogenesis [21]. Another study also demonstrated that malpar1 KO mice, which display enhanced oxidative stress in the hippocampus, showed abnormalities of hippocampal neurogenesis [22]. Catalase-overexpressed mice show improved neurogenesis and neuroprotective effects against radiation-induced degradation of hippocampal neurogenesis [23]. Peroxiredoxins (PRDXs) are also well studied in neural progenitor cell differentiation. Cytoplasmic PRDX1 and PRDX2 actively participate in the maintenance of embryonic stem cell stemness by opposing ROS/JNK activation during neurogenesis [24]. PRDX4 ablation causes premature neuronal differentiation and progenitor depletion [25]. Unlike other PRDXs, the role of PRDX6 in neurogenesis has not been reported yet. However, many of our studies have demonstrated that PRDX6 overexpression may even accelerate the development of AD, PD, and (experimental autoimmune encephalomyelitis) EAE [26–28]. Thus, we could speculate that PRDX6 may impair neurogenesis in these accelerating processes.

Several signaling pathways regulated by antioxidant enzymes have been reported for the control of neurogenesis. Nrf2-driven heme oxygenase 1 associated with Wnt/β-catenin signaling was causally related with the impairment of proliferation and differentiation of neural precursor cells [29]. A report suggested that excessive NO generation through the activation of CREB pathway modulates neuronal differentiation and development [30]. Several studies suggested that these critical pathways are regulated by TRIF to toll-like receptor 4 (TLR4) pathway. Moreover, TLR4 induces CREB signaling, which regulates early survival, neuronal gene expression, and morphological development in adult subventricular zone neurogenesis [31]. TLRs have recently been documented to be implicated in neurogenesis in mammalian [32] brain development [33]. TLR2 and TLR4 are found on adult neural stem/progenitor cells and have distinct and opposing functions in neural stem cell proliferation and differentiation, both in vitro and in vivo [32]. TLR2 deficiency in mice leads to impaired hippocampal neurogenesis, whereas the absence of TLR4 resulted in enhanced proliferation and neuronal differentiation [32]. In vitro studies further indicated that TLR2 and TLR4 directly modulated self-renewal and the cell-fate decision of NPCs. These data indicate that the TLR pathway and TLR-associated signaling pathway could be significant in controlling oxidative stress-induced neurogenesis.

We identified that PRDX6 overexpression impaired neurogenesis, and several factors were changed in the neural stem cells isolated from PRDX6 Tg mice. Among them, compared with wild-type mice, neural stem cells isolated from PRDX6 Tg mice show a dramatic decrease in WDFY1. We have identified WD-repeat- and FYVE-domain-containing protein 1 (WDFY1) as located downstream of the Neuropilin 2 (NRP2) axis [34]. WDFY1 is involved in placental development and for the maintenance of hematopoietic stem cells [35]. Interestingly, WDFY1 has been shown to recruit the signaling adaptors TLR3 and TLR4 [36], thereby potentiating the signaling necessary for neural stem cell differentiation. Although WDFY1 is suggested to have a role in the development of neural stem cells, its role on PRDX6-induced neurogenesis and the possibly involved mechanisms remain unclear. Therefore, we investigated the role of PRDX6 in the control of neural stem cell differentiation and the involved action mechanisms using PRDX6 Tg mice.

## Results

### Effect of PRDX6 on the Differentiation of Neural Stem Cells

**Fig. 1:**
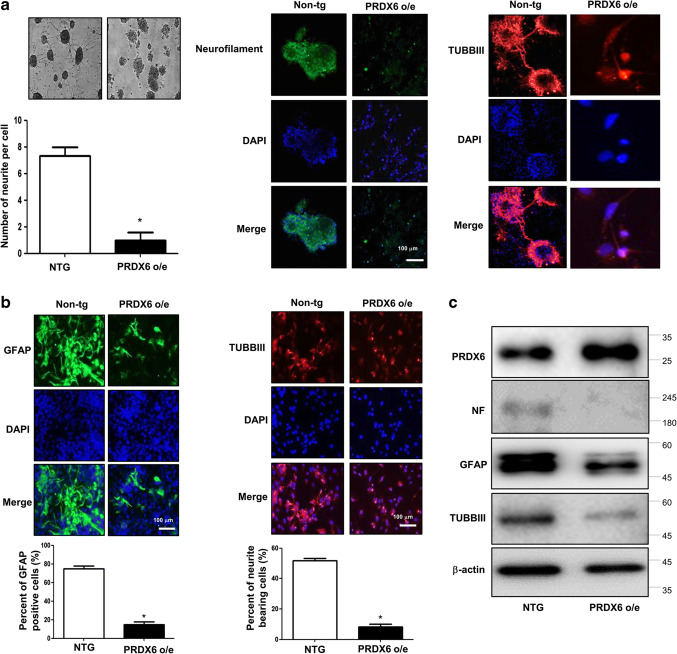
Effect of PRDX6 on the differentiation of neural stem cells. **a** Neural stem cells were isolated from embryonic day 15 forebrain germinal zones from Non-tg or PRDX6 Tg mice. Bulk cultures were established in the complete medium, then after 24 h, the medium was changed with neurobasal medium containing supplements. The cultured cells formed neurospheres at 5 days in vitro. The data are expressed as the mean ± SD of three experiments. *P < 0.05 indicates significant difference from control neural stem cells. **b** Neural stem cells were isolated, then differentiated into astrocytes and neuronal cells in vitro as described in materials and methods. Differentiated astrocyte or neuronal cells were stained with GFAP or TUBBIII (**b**). GFAP-positive cells and TUBBIII-positive neurite bearing cells were quantified. The data are expressed as the mean ± SD of three experiments. *P < 0.05 indicates significant difference from control neural stem cells. **c** Western blot analysis confirmed the expression of beta III-tubulin, GFAP, NF, and PRDX6 in terminally differentiated neurons and astrocytes. β-actin was internal control

To study the role of PRDX6 on the spontaneous differentiation of neural stem cells, we cultured primary neural stem cells isolated from the cortex of E15 day mouse embryos of wild-type or PRDX6 Tg mice. The cultured cells formed neurospheres at 5 days in vitro. We showed that neural stem cells from PRDX6 Tg mice resisted spontaneous differentiation for a prolonged time. PRDX6 Tg mice showed impaired differentiation and were unsuccessful in forming a high-quality network of neural cells. We observed that compared with wild types, stem cells from PRDX6 Tg had significantly shorter length of primary and secondary neurites as well as fewer neurites per cell (Fig. 1a, left panel). We also showed that the immunostained NF, which is a major component of the neuronal cytoskeleton and TUBBIII (a neuronal cell marker), was decreased in neural stem cells isolated from PRDX6 Tg mice (Fig. 1a, right panel). To understand the contribution of PRDX6 on the differentiation of neural stem cells into the specific cell types, we determined specific neuronal cell differentiation. We found that differentiated GFAP-positive glial cells and TUBBIII-positive neuronal cells were markedly fewer in PRDX6 Tg mice (Fig. 1b). We also showed decreased expression of NF, TUBBIII, and GFAP in PRDX6 Tg mice (Fig. 1c).

### Effect of PRDX6 on the Neurite Outgrowth of PC12 Cells

To check if the effect of PRDX6 on cell differentiation was similar in non-stem cells, PC12 cells were differentiated for 5 days upon stimulation with NGF (100 ng/mL) after the introduction of PRDX6 plasmid. PC12 has been previously used as an instructive model for studying the underlying the neuronal differentiation in response to NGF [37]. We showed that neurite outgrowth and branching of PC12 cells were stimulated by treatment with NGF, and this effect was inhibited by treatment with PRDX6 plasmid. In quantified data, the average number of neurites per cell was much lower in PRDX6 plasmid-treated cells than in control cells (Supplementary Fig. 1, left panel). The immunostained NF and TUBBIII were decreased in PRDX6-overexpressed PC12 cells (Supplementary Fig. 1, right panel).

### Involvement of WDFY1 in the Differentiation of Neural Cell Lineage

**Fig. 2:**
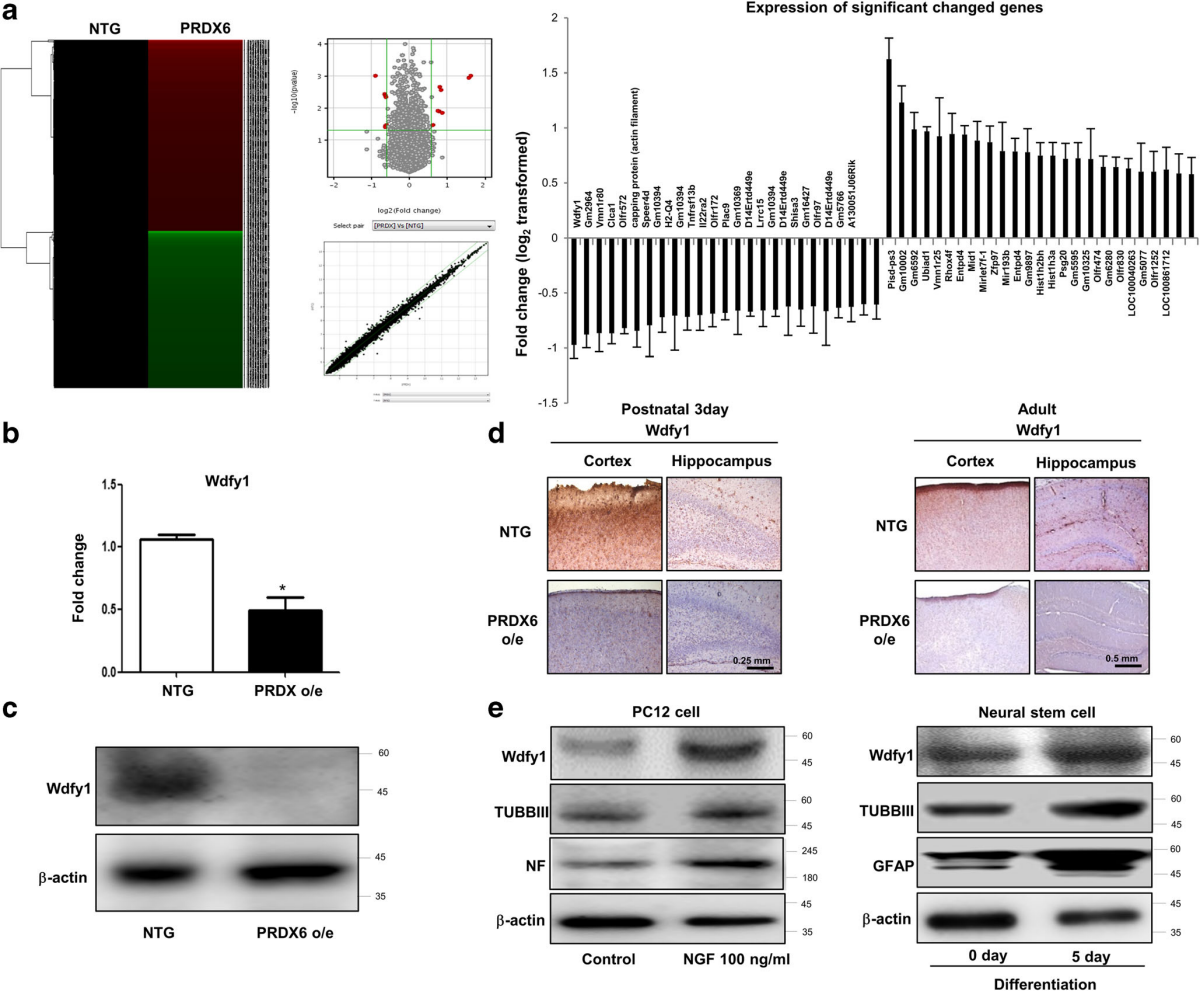
**a** Global gene expression profiles in the neural stem cells isolated from PRDX6 Tg mice. **a** Total RNA was obtained from the neural stem cells after 5 days of differentiation. Affymetrix GeneChip Mouse Gene 2.0 ST arrays containing 770, 317 mouse gene probes were used. Scatter plots show normalized intensities of each probe. **b** The expression of WDFY1 mRNA was determined by qPCR. **c** Western blotting. **d** Paraffin sections of brain from PRDX6 Tg or non-tg mice were stained with antibody against PRDX6. The data are expressed as the mean ± SD of three experiments. *P < 0.05 indicates significant difference from vector transfected neural stem cells. **e** PC12 cells were differentiated for 5 days upon stimulation with NGF (100 ng/ml) and neural stem cells were differentiated for 5 days; after that, we performed western blotting with WDFY1, TUBBIII, and NF antibodies

**Fig. 3:**
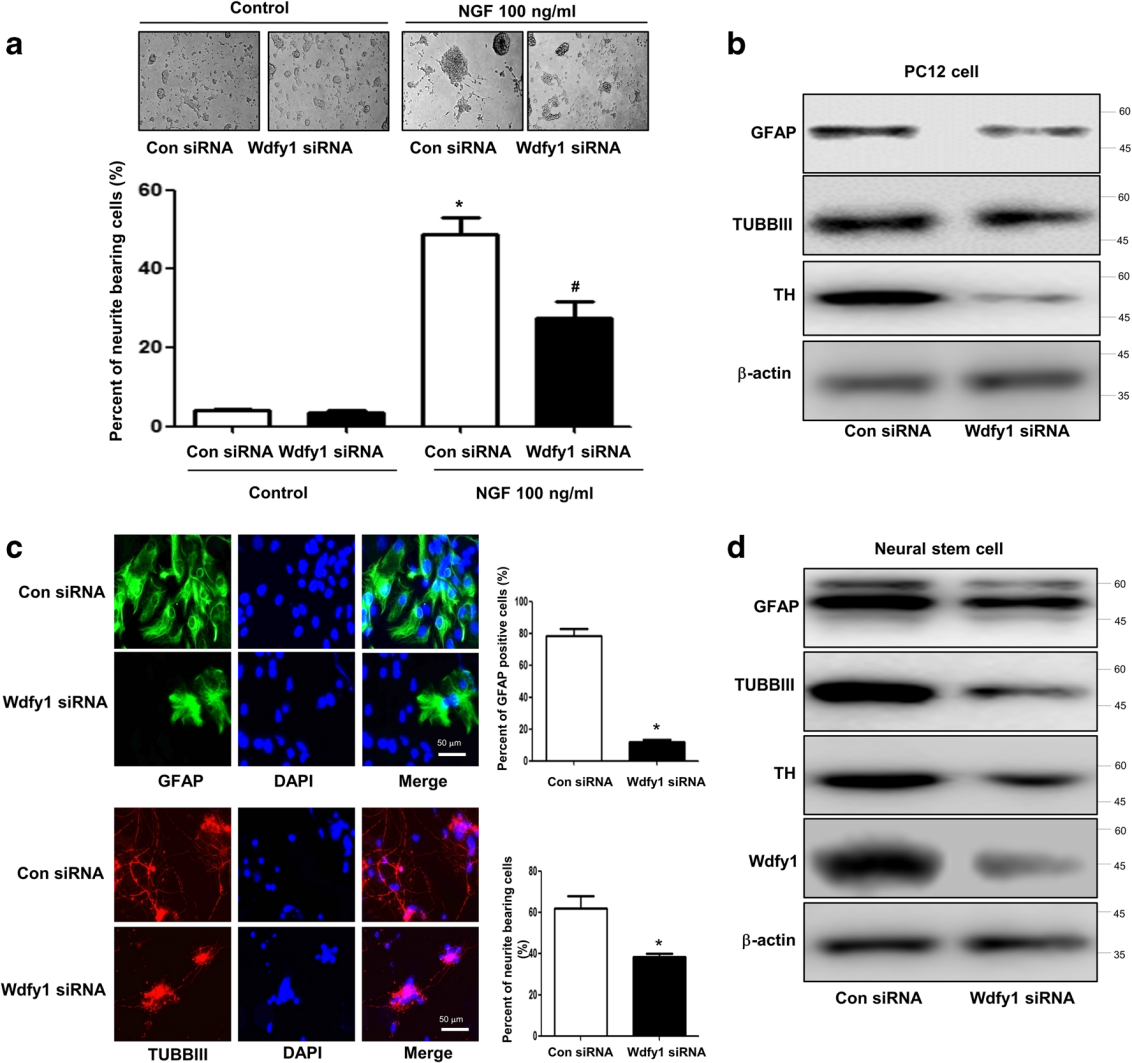
Effect of knockdown of WDFY1 on the differentiation of neural stem cells. **a** PC12 cells were differentiated for 5 days upon stimulation with NGF (100 ng/ml) after introduction of WDFY1 siRNA (100 nM). To study neurite outgrowth, the medium was changed to RPMI containing 100 ng/ml NGF. The cells were further cultured for 5 days. Cells with at least one neurite longer than two-body length were counted as neurite positive. At least 500 cells were counted for each group performed in triplicate. *P < 0.05 indicates significant difference from control siRNA-transfected NGF-non-treated PC12 cells. ^#^*P* < 0.05 indicates significant difference from control siRNA-transfected NGF-treated PC12 cells. **b** PC12 cells were transfected with WDFY1 siRNA and then differentiated for 5 days upon stimulation with NGF (100 ng/ml); after that, we performed western blotting with WDFY1, TUBBIII, NF and GFAP antibodies. β-actin was internal control. **c** Neural stem cells were transfected with WDFY1 siRNA for 48 h, then differentiated into astrocytes and neuronal cells in vitro as described in the “Materials and Methods” section and then immunostained with GFAP or TUBBIII antibodies. GFAP-positive cells and TUBBIII-positive neurite-bearing cells were quantified. The data are expressed as the mean ± SD of three experiments. *P < 0.05 indicates significant difference from control siRNA transfected neural stem cells. **d** Neural stem cells were transfected with WDFY1 siRNA and then differentiated for 5 days; after that, we performed western blotting with WDFY1, TUBBIII, NF, and GFAP antibodies. β-actin was internal control

**Fig. 4:**
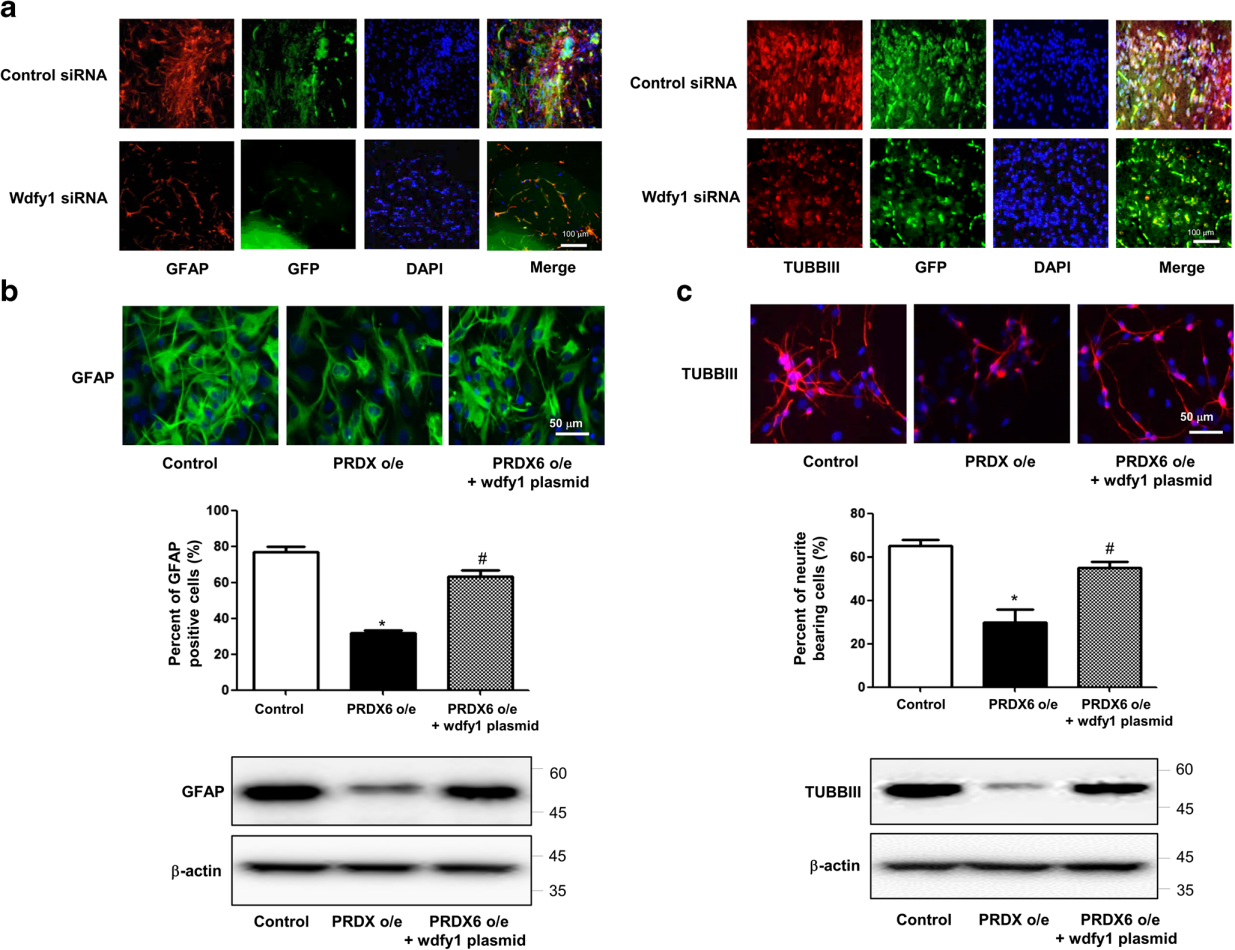
Effect of WDFY1 on the PRDX6-induced impaired differentiation of neural stem cells. **a** Neural stem cells transfected with WDFY1 siRNA, and then injected in SVZ region of ICR mice. After 2 weeks, differentiated astrocyte or neuronal cells were stained with GFAP or TUBBIII. **b**, **c** Neural stem cells isolated from non-tg or PRDX6 Tg mice were transfected with WDFY1 plasmid. Then, neural stem cells were differentiated into neuronal cells (**b**) and astrocytes (**c**) in vitro as described in the “Materials and Methods” section and then immunostained with beta III-tubulin or GFAP antibodies. β-actin was internal control. GFAP-positive cells and TUBBIII-positive neurite-bearing cells were quantified. The data are expressed as the mean ± SD of three experiments. *P < 0.05 indicates significant difference from vector-transfected neural stem cells. ^#^*P* < 0.05 indicates significant difference from PRDX6 transfected neural stem cells

To determine what the factors involved in PRDX6-related neural stem cell impairment, we performed a microarray experiment. In microarray data, several factors appeared to have changed. Among them, WDFY1 is dramatically changed in PRDX6 Tg mice-derived neural stem cells (Fig. 2a). Even though other factors, such as Gm2964, Vmn1r80, Clca1, Pisd-ps3, and Gm10002, were greatly changed, siRNA study of these factors did not reveal an effect on neurogenesis (data not shown). In addition, we confirmed that WDFY1 expression was dramatically decreased in PRDX6 Tg mice using RT-PCR (Fig. 2b), Western blotting (Fig. 2c), and immunohistochemistry (Fig. 2d). Few studies on the role of WDFY1 in neurogenesis have been reported. Thus, first, we demonstrated that WDFY1 expression was increased in PC12 cells after treatment with NGF and in neural stem cells after differentiation (5 days) (Fig. 2e). Next, we found that neurite outgrowth and branching of PC12 cells were stimulated by the treatment with NGF, and this effect was inhibited by treatment with WDFY1 siRNA. According to quantified data, average number of neurites per cell was much lower in WDFY1 siRNA-treated cells than in control cells (Fig. 3a); this was accompanied by reduced expression of GFAP, TUBBIII, and TH in the former (Fig. 3b, left panel). We also observed that WDFY1 siRNA decreased the ability of neural stem cells to differentiate into astrocytes and neuronal cells isolated from PRDX6 Tg mice (Fig. 3c); this was accompanied by reduced expression of GFAP, TUBBIII, and TH (Fig. 3d). Next, we performed an experiment to show the differentiation ability of WDFY1 in vivo. WDFY1 siRNA-transfected neural stem cells or EV control (30,000 particles in 1 mL; four animals per condition) were stereotaxically injected into the dorsal horn of the SVZ region of 10-week-old ICR mice using the following coordinates: AP + 0.5 and ML + 1.1 from the bregma and DV − 1.9 from the pial surface. Mice were euthanized 2 weeks following injection. On immunohistochemical staining of the mice brain, differentiated neuronal cells and astrocytes were found to be dramatically decreased by WDFY1 siRNA (Fig. 4a). Using immunofluorescence staining and Western blotting, we also found that impaired astrocytes (Fig. 4b) and neuronal cells (Fig. 4c) differentiated from neural stem cells in PRDX6 Tg mice were rescued by introduction of WDFY1 plasmid.

### Involvement of TLR Pathways in the WDFY1-Dependent Impairment of Neuronal Cell Differentiation in PRDX6 Tg Mice

**Fig. 5:**
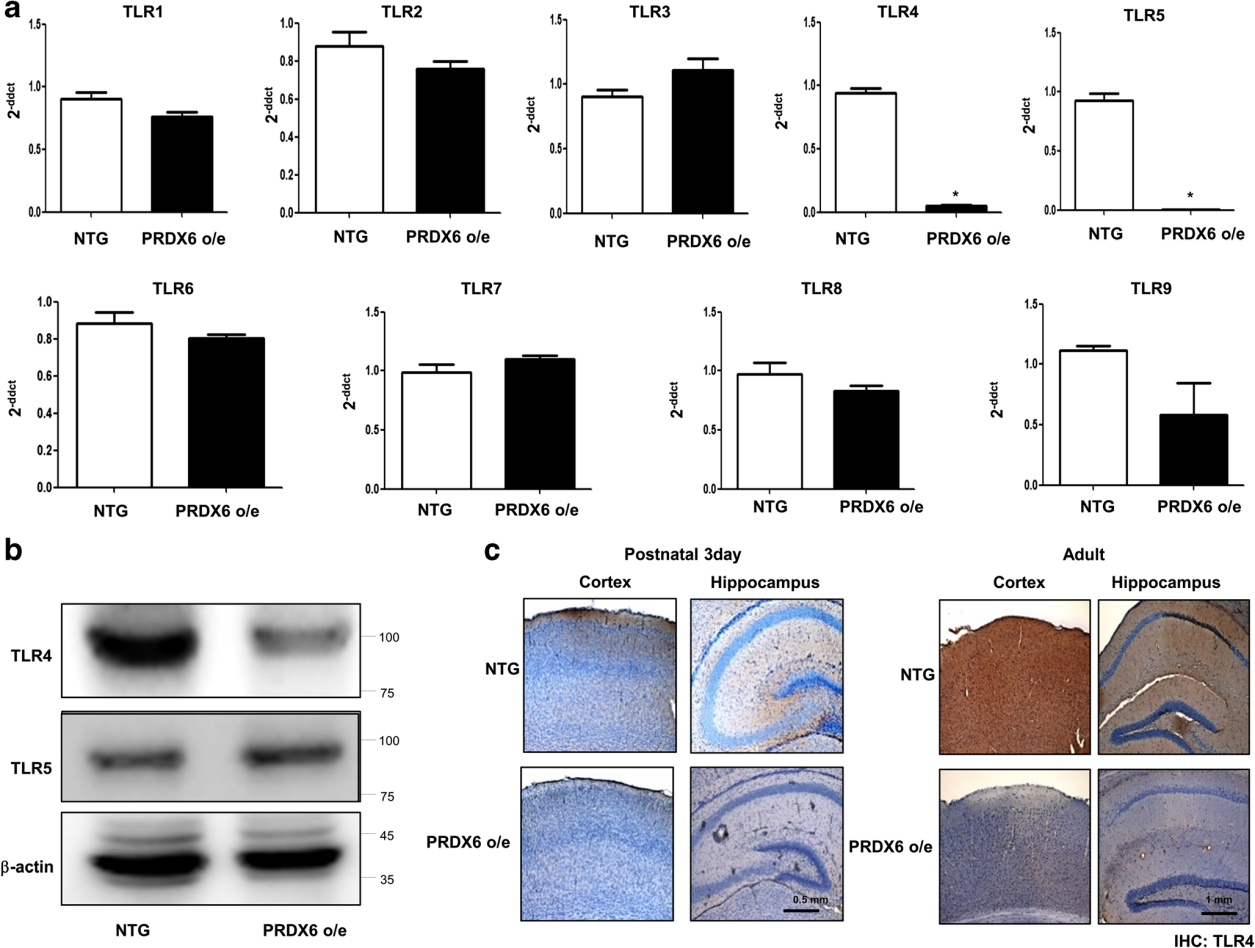
Effect of PRDX6 on the expression of TLR genes. Total RNA were obtained from the neural stem cells after 5 days of differentiation. Then, the expression of TLR1~TLR9 in neural stem cells was measured by qPCR (**a**) and Western blotting (**b**) and TLR4 by immunohistochemistry (**c**). The data are expressed as the mean ± SD of three experiments. *P < 0.05 indicates significant difference from control neural stem cells

**Fig. 6:**
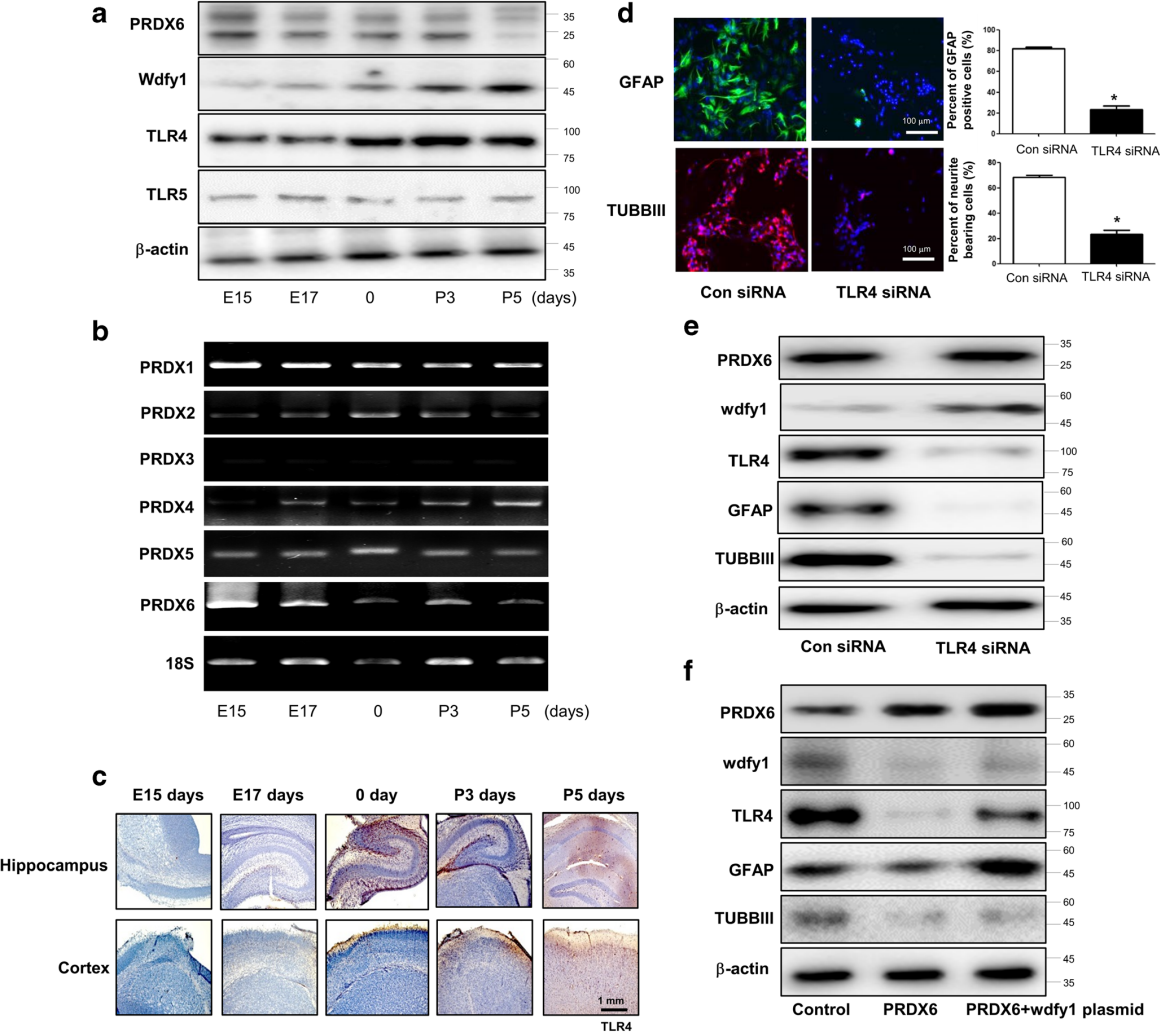
Expression pattern of TLR4 and WDFY1 on the development of brain of PRDX6 overexpressed mice. Brains were isolated at embryonic 15 days, embryonic 17 days, postnatal 0 day, postnatal 3 days, and postnatal 5 days, and then western blotting was performed with PRDX6, WDFY1, TLR4 and TLR5 antibodies (**a**), and RT-PCR was performed with PRDX1~6 specific primers (**b**). **c** Paraffin sections of brains were stained with antibody against TLR4 antibody. **d** Effect of knockdown of TLR4 on the differentiation of neural stem cells. Neural stem cells were transfected with TLR4 siRNA. Then, neural stem cells were differentiated into neuronal cells and astrocytes in vitro as described in the “Materials and Methods” section and then immunostained with GFAP or TUBBIII antibodies. GFAP-positive cells and TUBBIII-positive neurite-bearing cells were quantified. The data are expressed as the mean ± SD of three experiments. *P < 0.05 indicates significant difference from control siRNA-transfected neural stem cells. **e** Western blotted were performed with TLR4, GFAP, or TUBBIII antibodies. **f** Neural stem cells isolated from non-tg or PRDX6 o/e tg mice were transfected with WDFY1 plasmid. Then, neural stem cells were differentiated and then western blotted with TLR4, PRDX6, or WDFY1 antibodies. β-actin was internal control

We determined the involvement of TLR pathways in WDFY1-dependent PRDX6-induced neurogenesis impairment. Consequently, we found that PRDX6 was associated with TLR4 and WDFY1 through several factors in the GWAS data (Supplementary Fig. 2). In our study, among several TLR genes, expression of TLR4 and TLR5 was significantly decreased in neural stem cells in PRDX6 Tg mice compared with wild-type mice; however, the expression of other TLRs did not significantly change in PRDX6 Tg mice (Fig. 5a). We confirmed the expression pattern by Western blotting (Fig. 5b) and immunohistochemistry (Fig. 5c). We showed that TLR4 was dramatically reduced in PRDX6 Tg mice, but TLR5 was not expressed much. To determine whether the expression pattern of TLR4 depends on the development of brain, brains were isolated at day E15, day E17, postnatal day 0, and postnatal days 3 and 5, and Western blotting was then performed. We showed that the TLR4 expression increased depending on the development of brain and was accompanied with increases in WDFY1 but decreases in PRDX6 (Fig. 6a). In addition, using RT-PCR, we confirmed the expression of other PRDX subtypes. PRDX1 decreased marginally, PRDX2 and PRDX4 increased, PRDX3 was not detected, PRDX5 was unaffected, and PRDX6 dramatically decreased in a postnatal day-dependent manner (Fig. 6b). Moreover, we observed that TLR4 dramatically increased in a postnatal day-dependent manner by immunohistochemistry (Fig. 6c). Next, we showed the effect of TLR4 knockdown on the differentiation of neural stem cells. We showed that differentiated astrocytes and neuronal cells were reduced by introduction of TLR4 siRNA, as demonstrated by IF staining (Fig. 6d) and Western blotting (Fig. 6e). We also confirmed that the expression of differentiation markers, including TH, GFAP, and TUBBIII, was decreased by introduction of TLR4 siRNA in PC12 cell lines (Supplementary Fig. 3). We further found that reduced TLR4 expression was reversed by the introduction of WDFY1 plasmid (Fig. 6f). From these results, we suggest that TLR4 is critical for the development of neural stem cells and that this pathway is involved in the WDFY1-dependent impairment of neurogenesis in PRDX6 Tg mice.

## Discussion

Oxidative stress plays a major role in neuronal death caused by severe vulnerability of the brain to oxidation–reduction imbalance [38]. In the pathophysiology of neurodegenerative diseases, oxidative stress influences neurogenesis [17]. It has been demonstrated that the lack of extracellular SOD in the microenvironment impacts radiation-induced changes in neurogenesis [19]. Overexpression of other antioxidant enzymes, such as catalase and glutathione peroxidase, also increases neurogenesis, thus preventing neuronal injury. Overexpression of PRDX1, PRDX2, and PRDX4 is also associated with neurogenesis [24, 25]. Cytoplasmic PRDX1 and PRDX2 actively participate in the maintenance of embryonic stem cell stemness by opposing ROS/JNK activation during neurogenesis [24]. PRDX4 ablation causes premature neuronal differentiation and progenitor depletion [25]. We herein demonstrated that unlike other antioxidant enzymes, PRDX6 impairs neurogenesis. Our recent studies have demonstrated that PRDX6 accelerates the development of AD through increased amyloidogenesis [26]. We also recently found that PRDX6 Tg mice had a much higher loss of dopaminergic neurons, resulting in accelerated PD development [27]. Unlike other PRDXs, PRDX6 was dramatically decreased in a postnatal day-dependent manner. Thus, we speculated that impaired neurogenesis could contribute to the development of these neurodegenerative diseases. Even though it is possible that inducible phospholipase A2 (iPLA2) activity of PRDX6 could be associated with cell death and blocking of neurogenesis because iPLA2 is critical in the development of neurodegenerative diseases. However, its action mechanisms on neurogenesis impairment remain unclear.

To determine the factors involved in PRDX6-related neural stem cell impairment, we performed microarray experiments. From the resultant data, we identified that unlike in wild-type mice, in PRDX6 Tg mice, several factors were changed in the differentiated neural stem cells that were isolated. Among them, WDFY1 changed dramatically in PRDX6 Tg mice-derived neural stem cells. Even though other factors, such as Gm2964, Vmn1r80, Clca1, Pisd-ps3, and Gm10002, changed greatly, siRNA study of these factors showed no effect on neurogenesis (data not shown). In addition, we confirmed that WDFY1 expression was dramatically decreased in PRDX6 Tg mice. The role of WDFY1 was not fully understood because only few studies have been reported regarding the role of WDFY1 on neurogenesis. WDFY1 functions downstream of NRP2, which has an important role in gangliogenesis, axon guidance, and innervation in the sympathetic nervous system [39]. WDFY family has been associated with neurogenesis. Loss of wdfy3 in mice alters cerebral cortical neurogenesis, suggesting that WDFY3 is essential for cerebral expansion and functional organization [40]. Overexpression of WDFY1 reportedly abrogates early endosomal maturation, and thus hinders the fusion of autophagosomes with late endosomes for the formation of autolysosomes [34]. In the present study, we found that decreased neurogenesis in PRDX6 Tg mice was recovered by WDFY1 expression; however, WDFY1 knockdown inhibited NGF-induced PC12 cell differentiation and normal neurogenesis. Moreover, the WDFY1 siRNA-transfected neural stem cells stereotaxically injected into the dorsal horn of the SVZ region of 10-week-old ICR mice greatly reduced the differentiation into astrocytes and neuronal cells in the SVZ region of the brain. Moreover, the development of embryo (E15) into neonatal stages (post 3 days), the expression of WDFY1 was increased opposite to the PRDX6 expression. These data indicate that WDFY1 plays a significant role in the inhibition of neurogenesis in the brain of PRDX6 Tg mice.

It has been suggested that WDFY1 recruits the signaling adaptor TRIF to TLRs (TLR3 and TLR4) for the generation of several cytokines [36]. TLR proteins are present in mammalian brain cells in early embryonic stages of development and serve as negative modulators of early embryonic neural precursor cell proliferation and axonal growth [41]. Several members of the TLR pathway are more highly expressed in descending neurons (DNs) [42]. TLRs are also implicated in the modulation of neural precursor cell (NPC) differentiation. In addition, the effects of TLRs in the CNS also extend to the development of neuronal circuits. TLR2 deficiency diminished the number of neurons expressing early neuronal markers [43]. TLR3 is strongly expressed during early embryogenesis and decreases during the early postnatal period where it maintains a low expression [44]. Notably, neurite outgrowth is only affected by TLR3 activation; TLR3-deficient neurons do not exhibit augmented neurite outgrowth [44]. Activation of TLR8 by the synthetic ligand significantly reduces the length of primary neurites [45]. Only TLR8A is demonstrated to have a negative effect on neuronal viability; neuronal viability is not affected by activation of TLR3, TLR4, or TLR9 [45–47]. Therefore, the signaling pathway involved in the effect of TLR activation on neurite growth in neurons remains an open question. Interestingly, it has been demonstrated that PRDXs are involved in the TLR pathway; therefore, we investigated if there is a link between PRDX6 and WDFY1 via the TLR pathway for the control of neurogenesis in PRDX6 Tg mice. In the PRDX6 Tg mice brain, the expressions of TLR4, TLR5, TLR6, TLR8, and TLR9 were marginally decreased, whereas those of TLR3 and TLR7 were marginally increased. However, the expressions of only TLR4 and TLR5 were significantly decreased. On further study, TLR4 expression was found to be significantly increased in postnatal mice brain reversely consistent with PRDX6 expression. However, TLR5 expression did not change in developmental stages. Thus, we focused on TLR4 for further research. TLR4 knockdown significantly reduced neurogenesis, but PRDX6 expression was significantly increased. We further found that reduced TLR4 expression was reversed by introduction of WDFY1 plasmid, suggesting that TLR4 is critical for the development of neural stem cells and that this pathway is involved in the WDFY1-dependent impairment of neurogenesis in PRDX6 Tg mice.

Taken together, these studies indicated that PRDX6 inhibits neurogenesis of neural stem cells through downregulation of WDFY1-mediated TLR4 signaling pathway and suggest that the inhibitory effect of PRDX6 on neurogenesis plays a role in the development of neurodegenerative disease.

## Materials and Methods

### Animals and Isolation of Neural Stem Cells.

The PRDX6 Tg mice were purchased from the Jackson Laboratory (Bar Harbor, ME, USA). The non-transgenic and PRDX6 Tg mice used were WC57BL/6 mice. The mice were housed and bred under specific pathogen-free conditions at the Laboratory Animal Research Center of the Chungbuk National University, Korea (CBNUA-929-16-01). The mice were maintained in a room with a constant temperature of 22 ± 1 °C, relative humidity of 55 ± 10%, and under a 12/12-h light/dark cycle. Standard rodent chow (Samyang, Gapyeong, Korea) and purified tap water were available ad libitum. Neural stem cells were isolated from embryonic day 14 forebrain germinal zones from PRDX6 Tg or C57BL/6 (Jackson Laboratories). Bulk cultures were established, and the medium included DMEM/F12, 10% FBS, and 1% penicillin–streptomycin. After 24 h, the medium was changed to Neurobasal Medium (GIBCO, Germany) containing 1% glutamate, B27 supplement, 100 U/mL penicillin, and 100 μg/mL streptomycin.

### PC12 Cell Culture and Neurite Outgrowth

PC12 cells were obtained from the American Type Culture Collection (Manassas, VA, USA). RPMI1640, penicillin, streptomycin, fetal bovine serum (FBS), horse serum (HS), and nerve growth factor (NGF) were purchased from Invitrogen (Carlsbad, CA, USA). PC12 cells were grown in RPMI1640 with 5% FBS, 10% HS, 100 U/mL penicillin, and 100 μg/mL streptomycin at 37 °C in 5% CO_2_-humidified atmosphere.

### Neurite Outgrowth Assay

To study neurite outgrowth, the medium was changed to RPMI containing 1% HS, 100 ng/mL NGF, 100 U/mL penicillin, and 100 μg/mL streptomycin. The cells were further cultured for 5 days. Cells with at least one neurite longer than two-body length were counted as neurite positive. At least 500 cells were counted for each group; this was performed in triplicate.

### In Vitro Differentiation of Neural Stem Cells

For in vitro priming, neural stem cells were cultured in neurobasal medium plus B27 (Invitrogen), 0.1 mM 2-mercaptoethanol, 20 ng/mL b-fibroblast growth factor, 1 μg/mL laminin, 5 μg/mL heparin, 10 ng/mL nerve growth factor (Invitrogen), 10 ng/mL sonic hedgehog (R&D Systems), 10 μM forskolin (Sigma), and 1 μM retinoic acid (Sigma) for 5 days. For astrocyte induction, neural stem cells were cultured in poly-l-lysine-coated slides with DMEM medium plus N2 supplement and l-glutamate. All cultures were maintained in a humidified incubator at 37 °C and 5% CO_2_-humidified atmosphere, and half of the growth medium was replenished every third day.

### Transfection

PC12 cells were transiently transfected with pcDNA or PRDX6 plasmid (Santa Cruz Biotechnology) using the WelFect-EX Plus reagent in Opti-MEN, according to the manufacturer’s specification (WelGENE, Daegu, Korea).

### Immunofluorescence Staining

The fixed cells and tissues were permeabilized by exposure to 0.1% Triton X-100 for 2 min in PBS and were placed in blocking serum [5% bovine serum albumin (BSA) in PBS] at room temperature for 2 h. The cells and tissues were then exposed to a primary mouse monoclonal antibody for PRDX6 (1:200 dilution) and rabbit polyclonal antibody for Tubulin β-III (TUBBIII) (1:200) overnight at 4 °C. Then, they were washed with ice-cold PBS, followed by treatment with an anti-mouse secondary antibody labeled with Alexa Fluor 488 and anti-rabbit secondary antibody labeled with Alexa Fluor 568 (1:100 dilution, Molecular Probes Inc., Eugene, OR) for 2 h at room temperature. Next, immunofluorescence images were acquired using a confocal laser scanning microscope (TCS SP2, Leica Microsystems AG, Wetzlar, Germany) equipped with a × 400 oil immersion objective.

### Western Blot

The membranes were immunoblotted with primary specific antibodies. The blot was then incubated with corresponding conjugated anti-rabbit or anti-mouse immunoglobulin G-horseradish peroxidase (1:2000 dilution, Santa Cruz Biotechnology Inc.). Immunoreactive proteins were detected with the enhanced chemiluminescence Western blotting detection system. The relative density of the protein bands was scanned by densitometry using MyImage (SLB, Seoul, Korea) and quantified using Labworks 4.0 software (UVP Inc., Upland, CA, USA).

### Immunohistochemistry Staining

After transferring to 30% sucrose solutions, brains were cut into 20-μm sections using a cryostat microtome (Leica CM 1850; Leica Microsystems, Seoul, Korea). After two washes in PBS (pH 7.4) of 10 min each, endogenous peroxidase activity was quenched by incubating the samples in 3% hydrogen peroxide in PBS for 20 min and two washes in PBS of 10 min each. The brain sections were blocked for 1 h in 5% BSA solution and incubated overnight at 4 °C with a mouse polyclonal antibody against p21 (1:200; Santa Cruz Biotechnology, Inc., Santa Cruz, CA, USA), glial fibrillary acidic protein (GFAP) (1:200; Santa Cruz Biotechnology, Inc., Santa Cruz, CA, USA), and TUBBIII (1:200; Cell Signaling Technology, Inc., Beverly, MA, USA), and a rabbit polyclonal antibody against tyrosine hydroxylase (1:200; Cell Signaling Technology, Inc., Beverly, MA, USA). After incubation with these primary antibodies, brain sections were washed thrice in PBS for 10 min each. After washing, they were incubated with the biotinylated goat anti-rabbit or goat anti-mouse IgG-horseradish peroxidase (HRP) secondary antibodies (1:500; Santa Cruz Biotechnology, Inc., Santa Cruz, CA, USA) for 1 h at room temperature. Brain sections were washed thrice in PBS for 10 min each and were visualized using the chromogen DAB (Vector Laboratories) reaction for up to 10 min. Finally, brain sections were dehydrated in ethanol, cleared in xylene, mounted with Permount (Fisher Scientific, Hampton, NH), and evaluated on a light microscope (Olympus, Tokyo, Japan) (× 50 or × 200).

### Immunofluorescence Staining

For immunofluorescence staining, the fixed cells and brain sections were exposed to TUBBIII (1:200; Cell Signaling Technology, Inc., Beverly, MA, USA) and neurofilament (NF; 1:200; Cell Signaling Technology, Inc., Beverly, MA, USA) primary antibodies for 2 h at room temperature. After incubation, the cells were washed twice with ice-cold PBS and incubated with an anti-mouse or mouse secondary antibody conjugated with Alexa Fluor 488 or 568 (Invitrogen-Molecular Probes, Carlsbad, CA) for 1 h at room temperature. Immunofluorescence images were acquired using an inverted fluorescent microscope Zeiss Axiovert 200 M (Carl Zeiss, Thornwood, NY) (× 200).

### Neural Stem Cell SVZ Injections

WDFY1 siRNA-transfected neural stem cells or EV control (30,000 particles in 1 mL, four animals per condition) were stereotaxically injected into the dorsal horn of the subventricular zone of 10-week-old ICR mice using the coordinates AP + 0.5 and ML + 1.1 from the bregma and DV − 1.9 from the pial surface. Mice were euthanized 2 weeks after injection, and the CNS tissue was fixed by transcardial perfusion with PBS followed by 4% paraformaldehyde. To count the cells in the olfactory bulb, five different fields at 203 were used from each animal.

### RNA Preparation

Total RNA was extracted from the mouse neural stem cells using TRI REAGENT (MRC, OH) according to the manufacturer’s instructions. Following homogenization, 1 mL of solution was transferred into a 1.5-mL Eppendorf tube and centrifuged at 12,000×*g* for 10 min at 4 °C to remove insoluble materials. The RNA-containing supernatant was collected, mixed with 0.2 mL of chloroform, and centrifuged at 12,000×*g* for 15 min at 4 °C. After the RNA in the aqueous phase was transferred into a new tube, it was precipitated by mixing with 0.5 mL of isopropyl alcohol and recovered by centrifuging the tube at 12,000×*g* for 10 min at 4 °C. The RNA pellet was briefly washed in 1 mL of 75% ethanol and was then centrifuged at 7500×*g* for 5 min at 4 °C. Finally, the total RNA pellet was dissolved in nuclease water, and its quality and quantity was assessed using Agilent bioanalyzer 2100. Gene expression was analyzed using GeneChip® Human Gene 2.0 ST Arrays (Affymetrix, Santa Clara, CA), which comprises over 21,000 protein coding transcripts and over 19,000 entrez genes. For each gene, 11 pairs of oligonucleotide probes are synthesized in situ on the arrays.

### Microarray

Fragmented and labeled single-stranded DNA (ss-DNA) was prepared according to the standard Affymetrix protocol from 400 ng total RNA (GeneChip® WT PLUS Reagent Kit Manual, 2001, Affymetrix). Following fragmentation, 3.5 μg of ss-DNA was hybridized for 16 h at 45 °C and 60 rpm on GeneChip® CHO Gene 2.0 ST Array. GeneChips were washed and stained in Affymetrix Fluidics Station 450. GeneChips were scanned using Affymetrix GeneChip Scanner 3000 7G. The data were analyzed by Robust Multichip Analysis using Affymetrix default analysis settings and global scaling as the normalization method. The trimmed mean target intensity of each array was arbitrarily set to 100. The normalized and log-transformed intensity values were then analyzed using GeneSpring GX 13.1 (Agilent technologies, CA). Fold-change filters included the requirement that the upregulated genes should be present in ≥ 200% of controls and downregulated genes should be present in < 50% of controls. Hierarchical clustering data were clustered groups that behave similarly across experiments using GeneSpring GX 13.1 (Agilent technologies, CA).

### Quantitative Real-Time PCR

For mRNA quantification, total RNA was extracted using easy-BLURTM total RNA extraction kit (iNtRON Biotech, Daejeon, Korea). cDNA was synthesized using High-Capacity cDNA Reverse Transcription Kits (Applied Biosystems, Foster city, CA) according to the manufacturer’s instructions. Briefly, 2 μg of total RNA was used for cDNA preparation. Quantitative real-time PCR (qPCR) was performed using Brilliatn III Ultra-Fast Green QPCR Master Mix (Agilent Technologies, Waldbronn, Germany) specific for 18S and WDFY1 (5′-ACCATCCGAGTATGGCTGAAA-3′ and 5′-CCTGCTGTCGTGGTGGTATG-3′). All reverse transcription reactions were run in a StepOnePlus Real-Time PCR System (Applied Biosystems, Foster city, CA) using the universal cycling parameters (3 min at 95 °C, followed by 40 cycles of 5 s at 95 °C and 12 s at 60 °C each). Results were normalized to 18S and quantified relative to the expression in control samples. For relative quantification calculation, the 2^−ΔΔCT^ formula was used, where −ΔΔ CT = (C_T, target_ − C_T,18S_) experimental sample − (C_T, target_ − C_T,18S_) control sample.

### Statistical Analysis

All statistical analysis was performed with GraphPad Prism 5 software (Version 5.03; GraphPad software, Inc., San Diego, CA). Data were analyzed by one-way analysis of variance (ANOVA) followed by Dunnett’s *post hoc* test or two-way ANOVA followed by Bonferroni *post hoc* test according to the experimental design. All values are presented as mean ± S.D. Significance was set at *p* < 0.05 for all tests.

### Electronic Supplementary Material

Supplementary Fig. 1 **Effect of PRDX6 on the differentiation of PC12 cells. A,** PC12 cells were differentiated for 5 days upon stimulation with NGF (100 ng/ml) after introduction of PRDX6 o/e plasmid. To study neurite outgrowth, the medium was changed to RPMI containing 100 ng/ml NGF. The cells were further cultured for 5 days. Cells with at least one neurite longer than two-body length were counted as neurite positive and immunostained with neurofilament and TUBBIII. At least 500 cells were counted for each group performed in triplicate. *P < 0.05 indicates significant difference from pcDNA transfected NGF-non-treated PC12 cells. ^#^*P* < 0.05 indicates significant difference from pcDNA transfected NGF-treated PC12 cells. The data are expressed as the mean ± SD of three experiments. *P < 0.05 indicates significant difference from vector transfected PC12 cells. ^#^*P* < 0.05 indicates significant difference from PRDX6 transfected PC12 cells. (PNG 820 kb)

High resolution image (TIF 6444 kb)

Supplementary Fig. 2 **Gene network analysis using GeneMANIA.** The relationships between PRDX6, WDFY1 and TLR4 are shown based on known functional association networks. (PNG 1263 kb)

High resolution image (TIF 7983 kb)

Supplementary Fig. 3 **Effect of knockdown of TLR4 on the differentiation marker of PC12 cells.** PC12 cells were transfected with TLR4 siRNA. Then Western blotted were performed with TLR4, TH, GFAP or TUBBIII antibodies. β-actin was internal control. (PNG 165 kb)

High resolution image (TIF 2343 kb)
