# Correction to: PRDX6 Inhibits Neurogenesis through Downregulation of WDFY1-Mediated TLR4 Signal

**DOI:** 10.1007/s12035-024-04353-8

**Published:** 2024-07-17

**Authors:** In Jun Yeo, Mi Hee Park, Dong Ju Son, Ji Young Kim, Kyoung Tak Nam, Byung Kook Hyun, So Young Kim, Myung Hee Jung, Min Ji Song, Hyung Ok Chun, Tae Hyung Lee, Sang-Bae Han, Jin Tae Hong

**Affiliations:** College of Pharmacy and Medical Research Center, Chungbuk National University, 194-31, Osongsaengmyeong 1-ro, Heungdeok-gu, Cheongju, Chungbuk 361-951 Republic of Korea

**Correction to: Molecular Neurobiology (2019) 56:3132–3144**

10.1007/s12035-018-1287-2

The original online version of this article was revised:

In Fig. 1a of this article, it was determined that the PRDX6 o/e image in the right panel was incorrectly included. The figure 1a should have appeared as shown below.
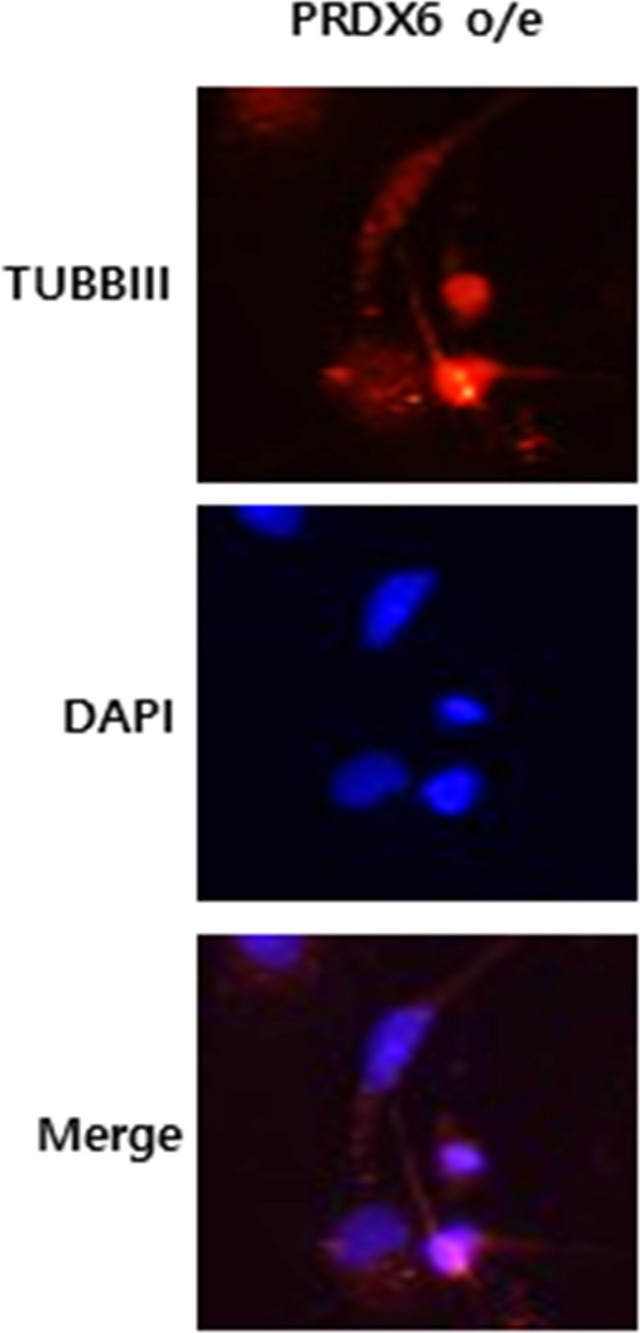


The original article has been corrected.

