# Supplementary figures and images for: PRDX6 Inhibits Neurogenesis through Downregulation of WDFY1-Mediated TLR4 Signal
Source: Mol Neurobiol. 2018 Aug 10;56(5):3132–44. doi: 10.1007/s12035-018-1287-2 (PMC6476867; doi:10.1007/s12035-018-1287-2)

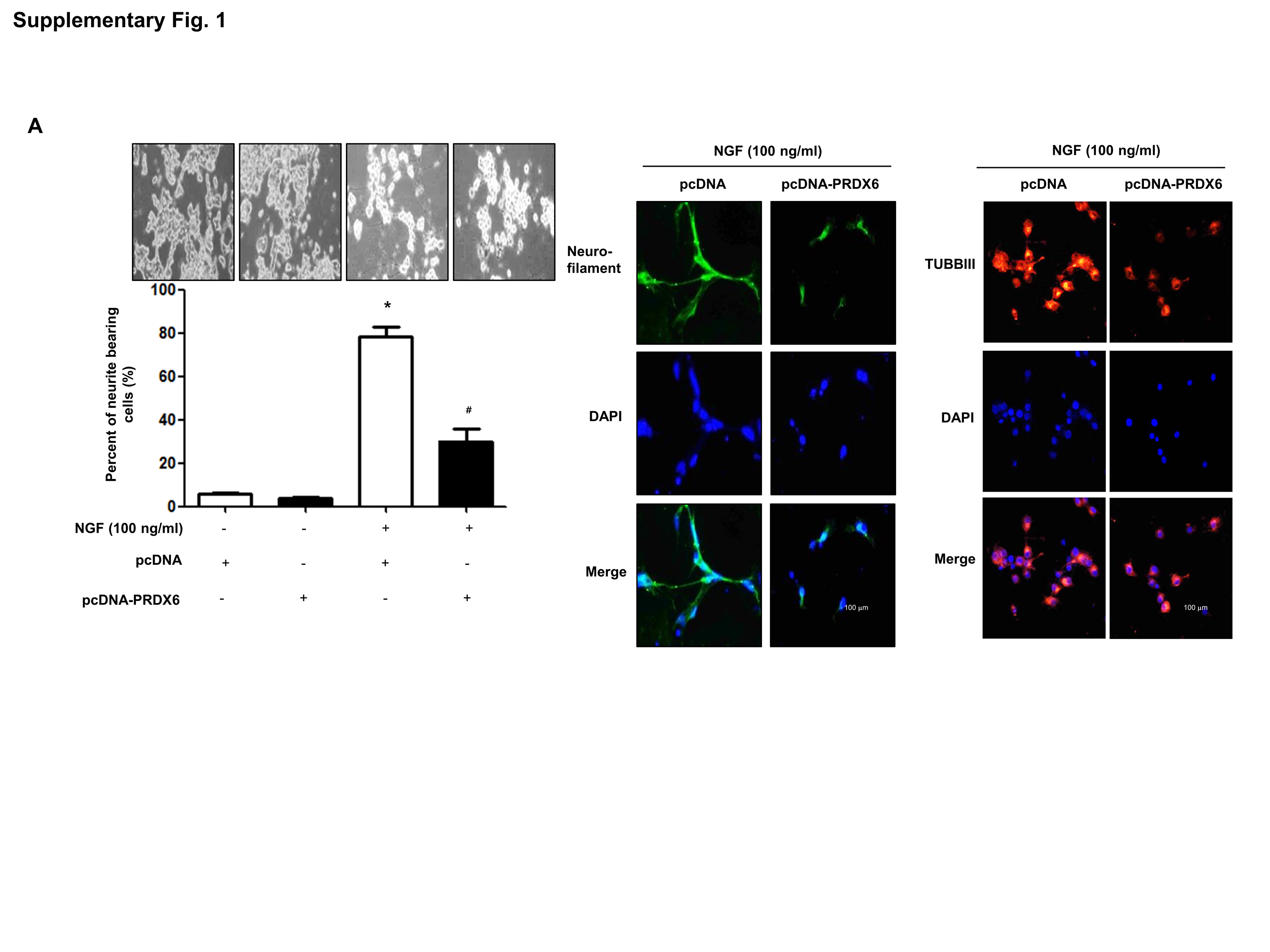

Supplement: Supplementary file 1 — Effect of PRDX6 on the differentiation of PC12 cells. A, PC12 cells were differentiated for 5 days upon stimulation with NGF (100 ng/ml) after introduction of PRDX6 o/e plasmid. To study neurite outgrowth, the medium was changed to RPMI containing 100 ng/ml NGF. The cells were further cultured for 5 days. Cells with at least one neurite longer than two-body length were counted as neurite positive and immunostained with neurofilament and TUBBIII. At least 500 cells were counted for each group performed in triplicate. *P < 0.05 indicates significant difference from pcDNA transfected NGF-non-treated PC12 cells. #P < 0.05 indicates significant difference from pcDNA transfected NGF-treated PC12 cells. The data are expressed as the mean ± SD of three experiments. *P < 0.05 indicates significant difference from vector transfected PC12 cells. #P < 0.05 indicates significant difference from PRDX6 transfected PC12 cells. (PNG 820 kb) [file 12035_2018_1287_Fig7_ESM.png]

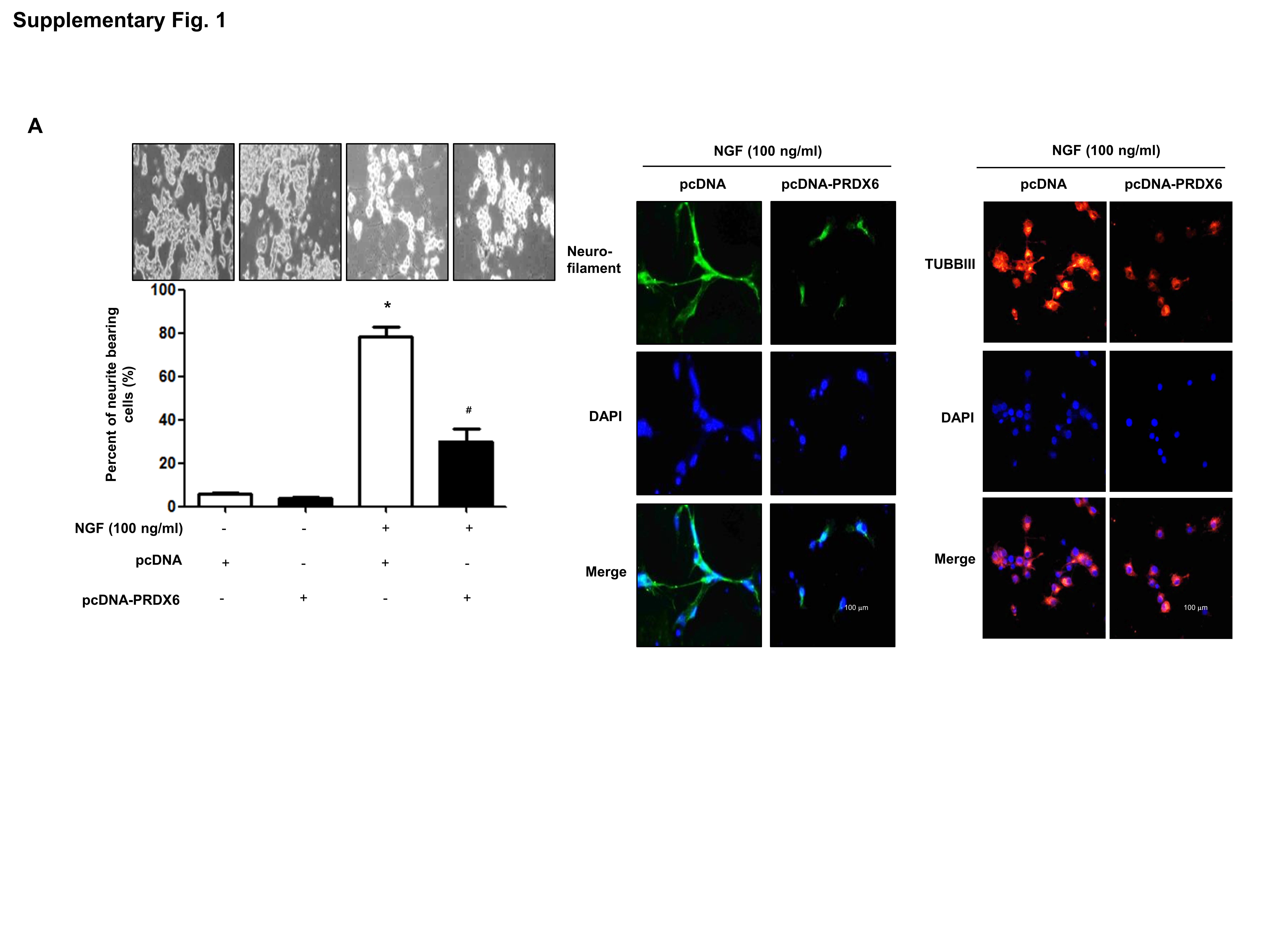

Supplement: Supplementary file 2 — High resolution image (TIF 6444 kb) [file 12035_2018_1287_MOESM1_ESM.tif]

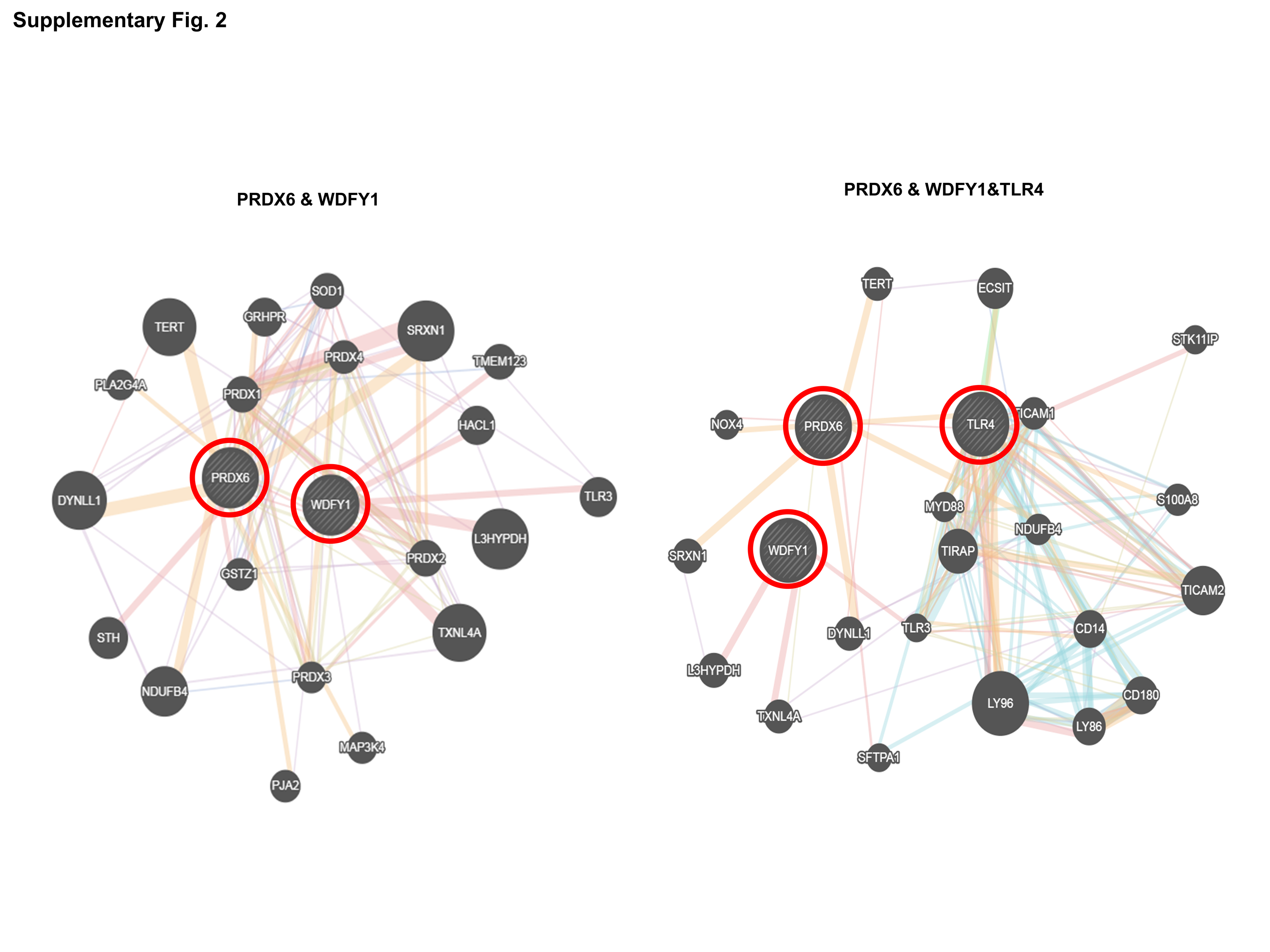

Supplement: Supplementary file 3 — Gene network analysis using GeneMANIA. The relationships between PRDX6, WDFY1 and TLR4 are shown based on known functional association networks. (PNG 1263 kb) [file 12035_2018_1287_Fig8_ESM.png]

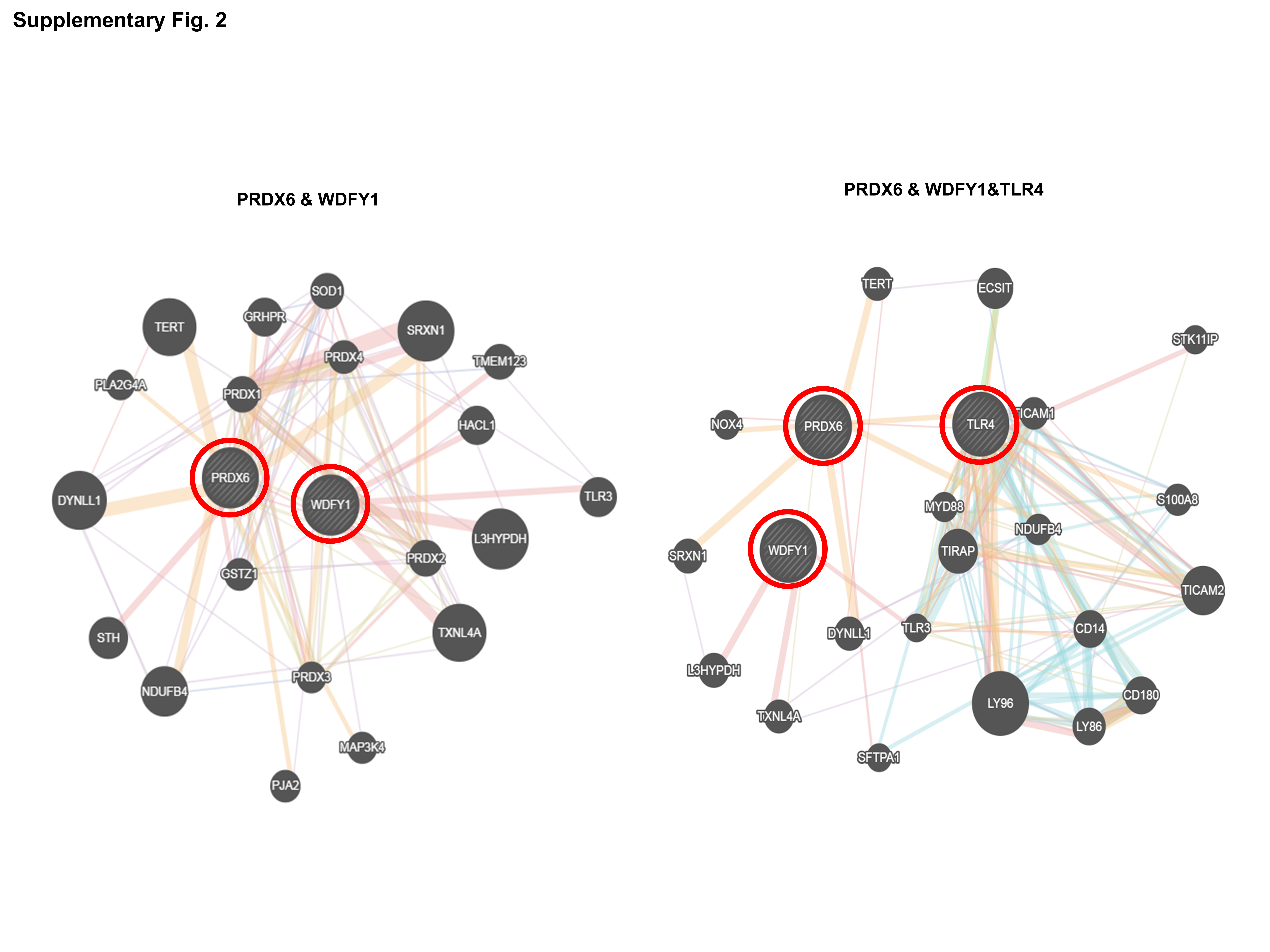

Supplement: Supplementary file 4 — High resolution image (TIF 7983 kb) [file 12035_2018_1287_MOESM2_ESM.tif]

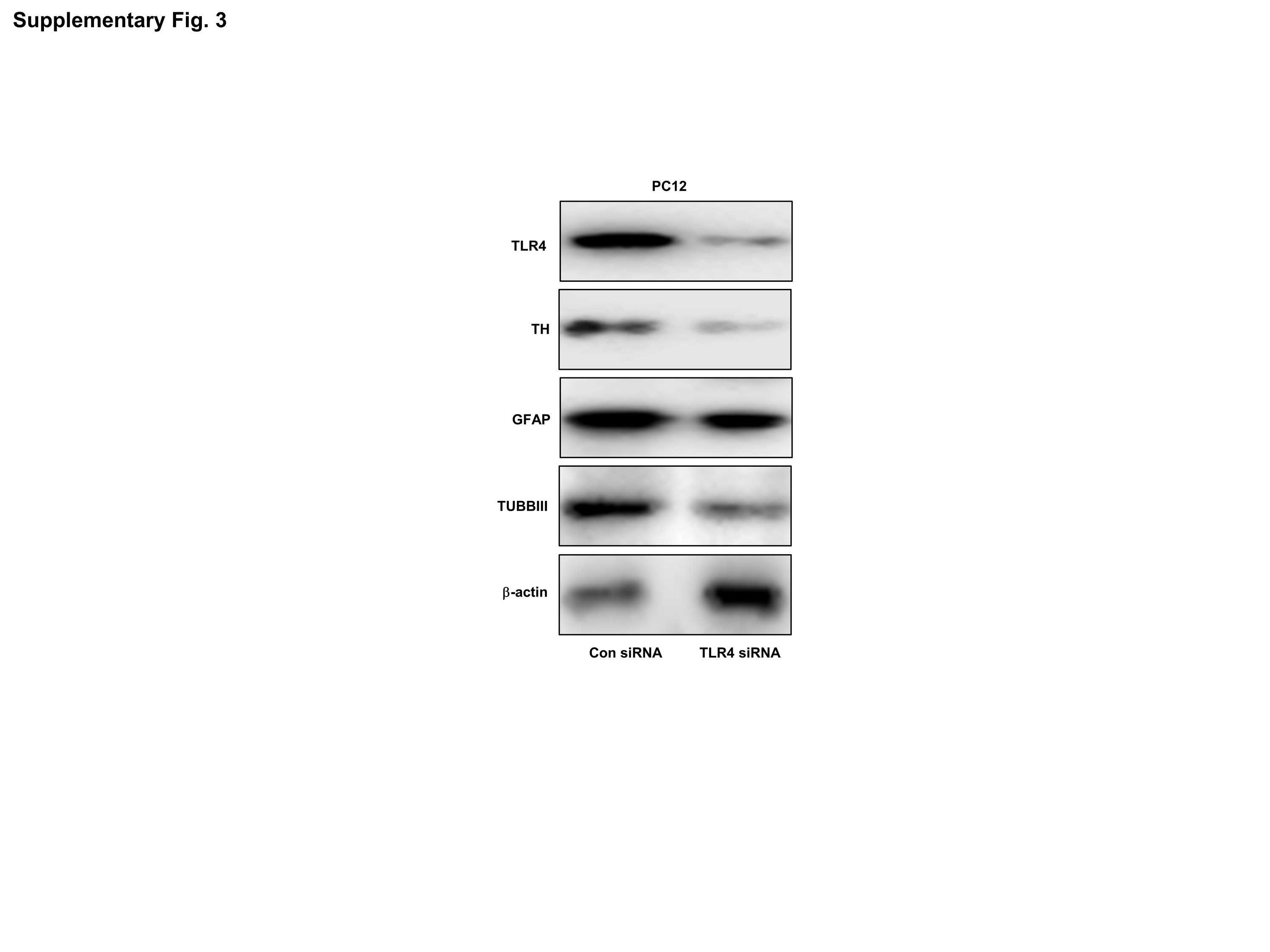

Supplement: Supplementary file 5 — Effect of knockdown of TLR4 on the differentiation marker of PC12 cells. PC12 cells were transfected with TLR4 siRNA. Then Western blotted were performed with TLR4, TH, GFAP or TUBBIII antibodies. β-actin was internal control. (PNG 165 kb) [file 12035_2018_1287_Fig9_ESM.png]

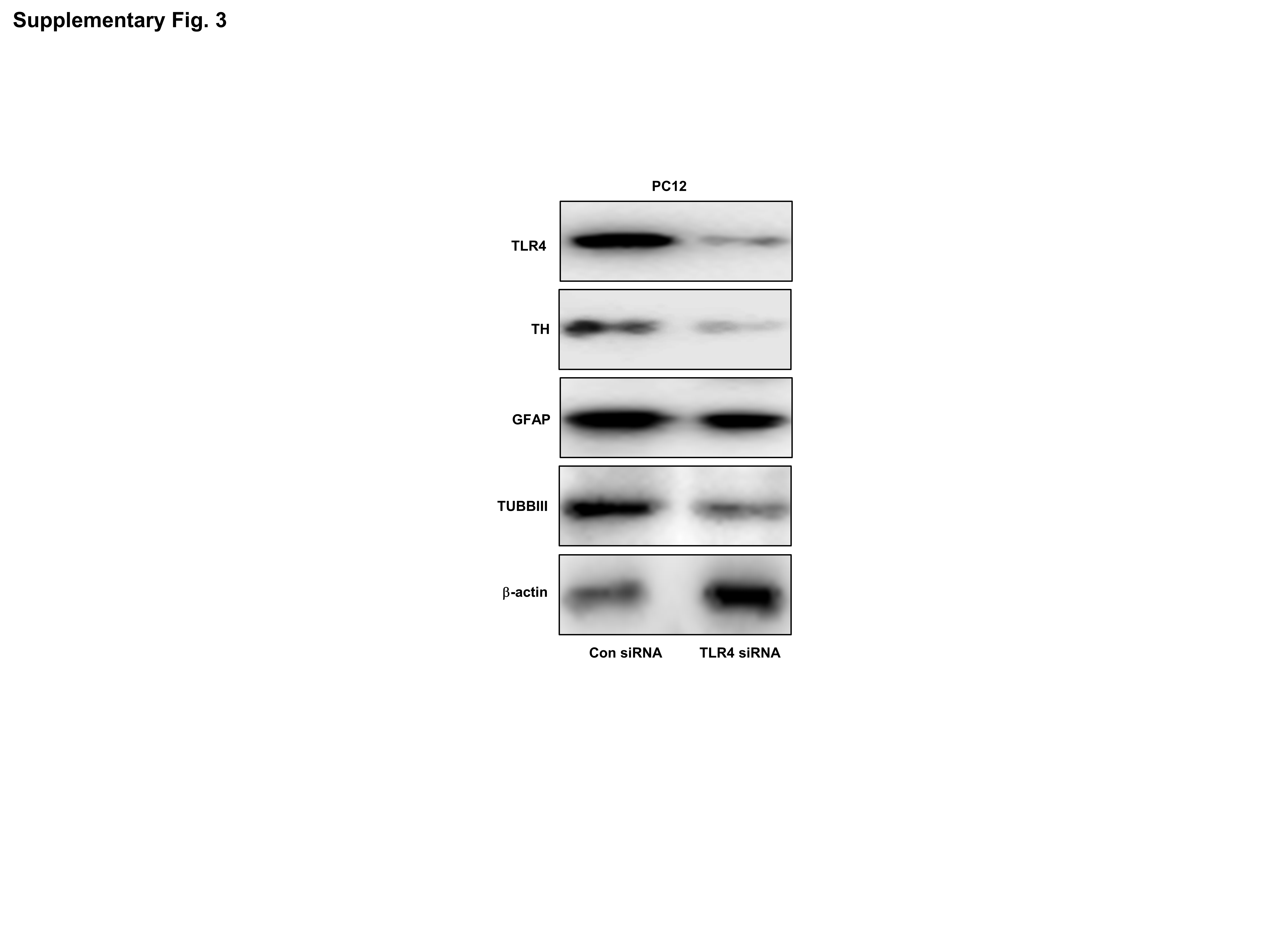

Supplement: Supplementary file 6 — High resolution image (TIF 2343 kb) [file 12035_2018_1287_MOESM3_ESM.tif]
